# Hearing Outcomes Following Endolymphatic Duct Blockage Surgery and Factors Associated with Improved Audition at Two Years Follow-Up

**DOI:** 10.3390/audiolres13030038

**Published:** 2023-06-02

**Authors:** Issam Saliba, Marc-Henri Asmar

**Affiliations:** 1 University of Montreal Hospital Centre (CHUM), Montreal, QC H2X 3E4, Canada; 2 University of Montreal Hospital Centre Research Centre (CRCHUM), Montreal, QC H2X 0A9, Canada; 3 Division of Otolaryngology-Head & Neck Surgery, University of Montreal, Montreal, QC H3C 3J7, Canada

**Keywords:** Ménière’s disease, endolymphatic sac, endolymphatic duct blockage, decompression, hearing, outcome

## Abstract

**Objective:** To evaluate hearing outcomes at 2 years post endolymphatic duct blockage (EDB) surgery, with an analysis of factors that may predict hearing improvement. **Study Design:** Retrospective comparative study. **Setting:** Tertiary care center. **Subjects:** Definite Ménière’s Disease (MD) patients undergoing EDB for refractory disease. **Methods:** Chart review was conducted to assign cases to one of the three hearing outcome groups (deteriorated, stable, and improved). All cases that met our inclusion criteria were selected. Preoperative data collected were audiograms, bithermal caloric tests, preoperative vertigo episodes, history of previous ear surgery for Ménière, intratympanic steroid injections (ITS) and intraoperative endolymphatic sac (ELS) tear or opening. Postoperative data collected at 24 months were audiograms, vertigo episodes and bithermal caloric testing. **Results:** Preoperative vertigo episodes, caloric paresis and history of surgery, ITS injections or ELS integrity, as well as postoperative vertigo class distribution and caloric paresis changes were not different between our groups. Preoperative word recognition score (WRS) was lowest in the improved hearing group (*p* = 0.032). The persistence of tinnitus at 2 years postoperatively was associated with deteriorated hearing (*p* = 0.033). **Conclusions:** There are no strong predictors of hearing improvement on presentation pre-EDB, but low preoperative WRS may be the best estimator available. Therefore, ablative interventions should be considered very carefully in patients presenting with low WRS, as they may benefit more from EDB; there is a fair chance of a good hearing outcome with EDB surgery. Persistence of tinnitus can reflect deteriorating audition. Vertigo control and hearing preservation are independent outcomes of EDB surgery, making it desirable as an early intervention for refractory MD cases.

## 1. Introduction

Ménière’s Disease (MD) is characterized by episodic vertigo, fluctuating sensorineural hearing loss, aural fullness, and tinnitus, with an incidence of 15–50 per 100,000 [1,2,3]. Low-frequency conductive hearing loss may be detected in Meniere’s disease, which is apparently not indicative of middle ear pathology. High incidence of conductive involvement in patients who have had a recent episode of vertigo may indicate a distortion of vibratory movement of the stapes [4].

Refractory MD has been challenging for otologists as there is no consensus on the efficacy of surgery to this day [4,5,6,7,8,9]. We have described a novel technique that controls symptoms with considerable success, supported by 10 years of experience: endolymphatic duct blockage surgery. It consists of a endolymphatic sac decompression followed by a crucial therapeutic step: blocking the endolymphatic duct with 2 titanium clips [10], thus separating the endolymphatic sac from the inner ear.

Surgery for refractory MD lacks evidence in the literature for many reasons: poorly designed trials, difficulty in establishing control groups, variable diagnostic criteria and different reporting methods. MD’s natural course further complicates outcome reporting: in the early years following the first vertigo episode, fluctuating hearing loss is more common as well as MD attacks. In the later stage of the disease, hearing loss stabilizes while vertigo becomes less frequent and may even subside completely. Unilateral and bilateral MD also affect hearing differently; unilateral MD has a more severe effect on low frequencies, while bilateral disease is less severe but more often affects higher frequencies [7,11,12,13].

Long term hearing outcomes are of particular interest with surgical treatment of MD because protecting patients from hearing loss over time has a considerable advantage, given that unlike vertigo, hearing loss is irreversible. In the literature, the different surgical techniques described result in a considerably wide range of outcomes: hearing improvement in 16–48% of cases [10,14,15,16,17,18,19,20] and hearing deterioration in 5.5–40% of cases [14,16,17,18,19,20,21,22], after at least 2 years of follow-up. It is important to note that many of these studies have serious flaws, as illustrated by Pullen et al.’s Cochrane article on surgery for MD, wherein only two trials in the literature qualified for meta-analysis [9].

Despite the lack of convincing evidence for surgical interventions, less invasive alternatives, such as medical management or intratympanic injections, do not protect patients from hearing loss associated with the disease. Many studies report even higher hearing deterioration rates in the ranges of 15.5–62% [8,21,23,24,25,26] and one study even suggests that all interventions have little to no effect on the progression of hearing loss [27].

The objective of this study is to evaluate hearing outcomes at 2 years post endolymphatic duct blockage (EDB) surgery, with an analysis of factors that may predict hearing improvement.

## 2. Materials and Methods

### 2.1. Study Design and Patient Population

This is a retrospective single physician study, conducted at our tertiary care center following approval from our institutional IRB. Patients with a clinical diagnosis of definite MD, according to the 1995 AAO-HNS criteria [28,29], and failure of medical therapy for at least 6 months opted for endolymphatic duct blockage surgery (EDB). Patient charts were consulted to determine inclusion for this study.

Inclusion criteria:

1. Diagnosis of definite MD;
2. EDB surgery performed at our institution by the same surgeon;
3. Age over 18 years old.

Exclusion criteria:

1. Incomplete records during the first 2 years post-EDB;
2. Bilateral Ménière’s Disease;
3. Disease onset more than 10 years before intervention;
4. Nonfunctional affected ear or “dead ear” on presentation.

All eligible EDB cases were considered and grouped according to hearing outcomes, defined by variations larger than 15 dB for PTA or 20% for WRS (deteriorated, improved, or stable). After applying our exclusion criteria, a total of 66 patients were included: 13 with deteriorated hearing, 15 with improved hearing and 38 with stable hearing.

### 2.2. Study Parameters

Demographic data included age and sex, reported as percentage of males. Preoperative parameters included a history of intratympanic steroid injections (ITS), a history of revision surgery, intraoperative ELS tear or opening, pure tone audiometry, vestibular caloric testing, aural fullness, tinnitus, and preoperative vertigo episodes (cumulative over 6 months). Postoperative parameters included pure tone audiometry, aural fullness, tinnitus, AAO-HNS vertigo class (Table 1) [28] and caloric paresis improvement.

Pure tone audiometry was performed on all patients at our tertiary care center’s audiology clinic. Average of hearing was calculated from hearing thresholds at 0.5, 1, 2 and 4 KHz and used for all analyses, including air conduction (PTA) and bone conduction (BCT). Word recognition scores (WRS) were recorded as the percentage of words repeated from a 25-word list at a comfortable hearing level. Audiometric testing was conducted the day before surgery and compared to the results obtained in the last 6 preoperative months; the worst audiogram was then selected for analysis. Postoperative testing was conducted at 2 years post-EDB. Vestibular caloric tests were performed in the 6 months before and during the first 6 months after EDB; bithermal testing was conducted for all cases at our institution. If the deficit affected the untreated ear, the value was reported as 0% for the operated ear.

Age, preoperative vertigo episodes, PTA, BCT, WRS and caloric paresis are reported as means [95% confidence interval]. Sex, ITS injections, revision surgery, ELS integrity and vertigo class are reported as proportions (% of cases).

### 2.3. Statistics

Statistical analysis was completed using SPSS version 24 (IBM, Chicago, IL, USA). The Shapiro–Wilk test was used to assess normality of each data set. One-way ANOVA was performed for continuous normally distributed variables. Intragroup testing for normal variables was conducted using paired T-tests. The Chi-square and McNemar tests were used for proportion distributions. For non-normally distributed data, the Kruskal–Wallis non-parametric test was performed, and intragroup testing was conducted using the Mann–Whitney test. *p* < 0.05 was considered statistically significant.

## 3. Results

### 3.1. Patient Demographics

Age and sex distributions are shown in Table 2. The stable, improved, and deteriorated hearing groups belonged to the same age group, with a mean 56.7, 53 and 50.7 years, respectively (*p* = 0.22). Sex distribution was also homogenous: 29% males in the stable hearing group, 43% males in the deteriorated hearing group and 50% in the improved hearing group (*p* = 0.343). Our groups were therefore adequately matched for analysis.

### 3.2. Hearing Outcomes

Our cohort as a whole showed stable hearing at 2 years post-EDB: mean postoperative PTA was 55.2 [49.9–60.6] dB (mean increase of 5.1 dB over 24 months); mean postoperative BCT was 46.5 [41.2–53.7] dB (mean increase of 0.8 dB over 24 months); and mean postoperative WRS was 62.9 [56.1–69.6%] (mean increase of 1% over 24 months). Neither of those changes are clinically significant. Table 2 details the outcomes for each group.

The deteriorated hearing group had a mean postoperative PTA of 69.4 [61.9–76.9] dB, BCT of 42.9 [55.1–66.3] dB and a mean WRS of 45.8 [35.2–56.4%]. Over 24 months, the mean PTA increase was 20.6 dB (*p* < 0.001) and the mean loss of word recognition was 25.7% (*p* < 0.001).

The stable hearing group had a mean postoperative PTA of 49.1 [39.9–58.4] dB, BCT of 42.9 [33.2–52.6] dB and a mean WRS of 63.4 [50.4–76.5%]. The mean increase over 2 years was 2.8 dB (*p* = 0.02), with a mean loss of 1.4% on word recognition (*p* = 0.3).

Finally, the improved hearing group had a mean postoperative PTA of 46.2 [37.3–55.1] dB, BCT of 39.5 [30.4–48.7] dB and a mean WRS of 80.1 [72.4–87.8%]. Over 2 years, the mean increase in word recognition was 28.3% (*p* < 0.001) while the PTA showed a mean loss of 8.6 dB (*p* = 0.002). 

**Figure 1:**
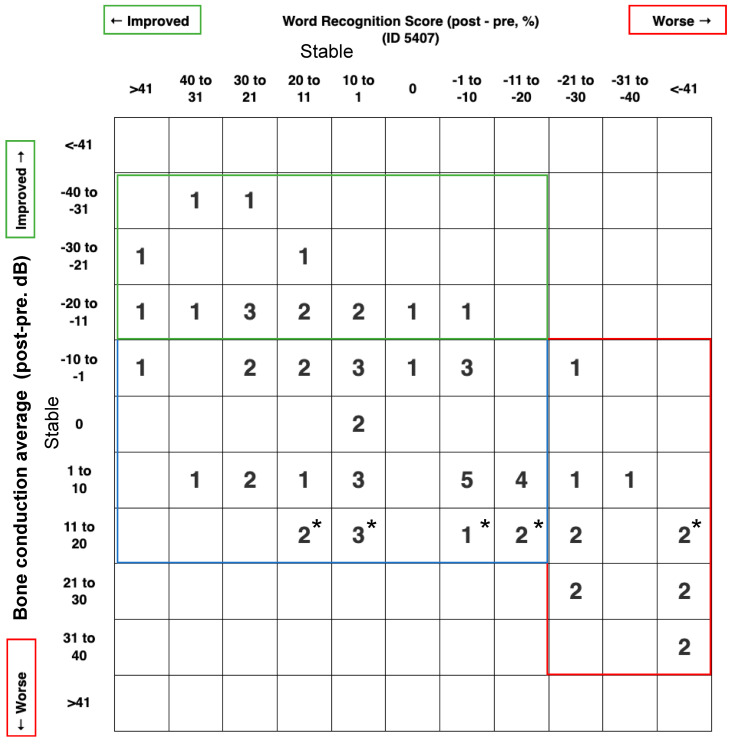
Scattergram 5407 for hearing outcome; post-pre treatment. This graph was generated according to AAO-HNS minimal reporting standards (http://hearingoutcomes.stanford.edu accessed on 9 April 2023) modified by using the bone conduction level instead of the pure tone average, since we are looking to analyze the effect of endolymphatic duct blockage on the inner ear cells. * means the bone conduction average is between 10 and 15 dB.

We also report a scattergram for these outcomes by using the BCT instead of the PTA to report the inner ear status. As shown in Figure 1, 13/66 patients (19.7%) suffered a deterioration of BCT or WRS, 15/66 patients (22.7%) improved their BCT and/or WRS, while 38/66 patients (57.6%) have both stable BCT and WRS.

### 3.3. Preoperative Factors

Preoperative factors are shown in Table 3. Histories of ITS injections, revision EDB surgery and intraoperative ELS integrity were not significantly different between our three groups (*p* = 0.236, *p* = 1, and *p* = 0.141, respectively). The cumulative number of vertigo spells in the 6 months before surgery was also similar between our groups (*p* = 0.17) with a mean 18 episodes in the stable hearing group, 32.14 in the improved hearing group and 28.15 in the deteriorated hearing group. Regarding bithermal caloric testing, preoperative caloric paresis was not significantly different between our groups (*p* = 0.29).

The difference observed in preoperative PTA between our groups was not statistically significant (*p* = 0.523). However, preoperative WRS was significantly lower in the improved hearing group when compared to the other two groups (*p* = 0.032).

### 3.4. Postoperative Factors

Postoperative factors are shown in Table 3. At 2 years post-EDB, our cohort had considerable improvement in vertigo episodes: 85% class A and 15% class B. We observed no statistically significant difference in the distribution among our groups: class A distribution was 76% with stable hearing, 91% with improved hearing and 87% with deteriorated hearing (*p* = 0.38).

Caloric paresis in the affected ears were not significantly changed after the intervention in any group, with mean improvements of 10.3% for all patients, 7.25% in the stable hearing group, 9.84% in the improved group and 11.4% in the deteriorated group (*p* = 0.22).

Regarding symptoms, persistence of aural fullness at 24 months was not associated with any particular group: 71.4%, 60% and 70% of cases in the stable, improved and deteriorated hearing groups, respectively (*p* = 0.647). However, persistence of tinnitus at 2 years was associated with a deteriorated hearing outcome: 76%, 82% and 100% of cases in the stable, improved, and deteriorated hearing groups, respectively (*p* = 0.033).

## 4. Discussion

### 4.1. Hearing Outcomes

The fluctuating nature of hearing loss associated with Ménière’s disease presents a challenge for adequate assessment of auditory function. In the literature, this is supported by the wide range of auditory outcomes reported following interventions [8,11,13,14,27,30,31,32]. In their study about auditory fluctuations in MD, Hoa et.al suggest that the problem with hearing as an outcome is the risk of mistaking treatment efficacy with temporary ameliorations [33]. On the other hand, hearing conduction impairment should not be considered as a middle ear problem, rather it is a high inner pressure decreasing the stapes vibration. Nonetheless, the best possible way to reconcile the variability in the literature and minimize bias is to report hearing outcomes uniformly, which is why the American Academy’s criteria are so important [28,29]. Long-term follow-up studies for large MD cohorts revealed that patients lose 1 dB per year on average, mostly before the age of 50, and that older subjects are less likely to deteriorate at this rate [11]. In our study, the patient population has a disease duration of under 10 years as a result of eligibility for surgery, which likely situates our cohort in the progressive disease phase: Hoa et. al’s observation was that progression to severe SNHL mostly occurred in the first 16.4 years [33]. Hearing outcomes post-EDB surgery favor stability of the bone conductive level (57.56%) or improvement (22,72%) in the first 2 years: this is desirable for an intervention that achieves class A vertigo control in 85% and class B in 15% of cases. Even though this study is clearly not equipped to detect a difference in the natural history of Ménière’s disease, we think these results of vertigo control are far from considering it as a natural evolution of the disease. Because BC reflects the inner ear status, we build our scattergram with the BCT instead of the PTA that reflects more the middle ear status, as suggested in the scattergram post middle ear surgery. Postoperative WRS is the measure that most clearly defines our groups with clinically significant changes from preoperative levels in both improved and deteriorated groups (+28.3% and −25.7%, respectively). Thus, WRS is a good measure of auditory function and should be taken into the account in the short term follow up.

We also note that mean PTA was not significantly different in our groups despite the difference in WRS; thus, PTA on presentation may not be a good predictor of outcome at 2 years. This is likely due to expected SNHL fluctuations in MD. Increased hydrops may not initially lead to cellular destruction which would be reflected by the PTA but does affect sound perception in the form of distortion, as suggested by the WRS differences observed. For a more complete picture, longer follow-up will be required to compare EDB’s auditory outcomes to other long-term reports in the literature.

### 4.2. Parameters Associated with Hearing Improvement

The ability to predict which hearing presentation is most likely to benefit from hearing improvement in MD after surgery would be an important asset to ENT surgeons: the decision to undergo EDB or other interventions, such as intratympanic Gentamycin injections or labyrinthectomy, can be better targeted. A long-term study for multiple procedures (medical treatment, endolymphatic mastoid shunt surgery and vestibular neurectomy) [27] concluded that PTA on presentation was more important than the type of intervention, and that poor hearing on presentation stabilized while good hearing deteriorated over a period of 20 years. In our study, we could not find that PTA was a good predictor of outcome at 2 years (*p* = 0.523). Instead, WRS on presentation was significantly lower in the improvement group (*p* = 0.032), suggesting that even where WRS is low (in the confidence interval [41.1–62.5]) there is a fair chance of a good hearing outcome. This is clinically relevant as poor functional hearing is often a reason to advocate a destructive procedure. Based on the data presented, we suggest ablative interventions, such as intratympanic Gentamycin injection, labyrinthectomy or vestibular neurectomy, should be considered very carefully with patients presenting with low WRS or even to be avoided, as they might benefit more from EDB. We can at least claim that EDB surgery is overall unlikely to have a severe negative impact on hearing. Long term outcomes cannot be extrapolated from this observation, as the fluctuating nature of hearing loss complicates this analysis. 

Regarding other preoperative factors, we could not find any that was particular to the improved hearing group. A positive history for intratympanic steroid injections or previous surgery for Ménière’s (at our institution, this includes lateral or triple semicircular canal obliteration and classic endolymphatic sac decompression) was not associated with any hearing outcome (*p* = 0.236, and *p* = 1, respectively). This suggests that hearing outcomes post-EDB are not affected by previous interventions and should not alter the management of EDB candidates. Furthermore, endolymphatic sac integrity during surgery, which can be difficult to maintain due to challenging dissection of the endolymphatic duct, has no impact on hearing outcomes (*p* = 0.141). This was reported too in 2016 by our team by using different data of patients [34]. Since no major complication was ever encountered with EDB, patients and physicians should have no cause for concern about the overall safety of the surgery. Finally, the number of preoperative vertigo spells as well as caloric paresis had no association with any hearing outcome (*p* = 0.17, and *p* = 0.29, respectively). The clinical implication is that hearing outcomes and symptoms control can be considered independently when evaluating a patient for surgery, making EDB an ideal procedure for refractory MD in the early disease phase. 

In the follow up period of 2 years post-EDB, we were also interested in postoperative parameters that could be associated with hearing improvement. Vertigo control was not particular to any group (*p* = 0.38), neither was any improvement in caloric paresis (*p* = 0.22). This is probably due to the very high rate of vertigo control irrespective of hearing outcome or functional status. Complete vertigo control (class A) and substantial control (class B) were achieved in 85% and 15%, respectively. Class A and B are considered as satisfactory control of vertigo and was obtained in 100% of cases at 2 years follow-up post-EDB. Since paresis of the vestibular function did not exceed 10% in the three groups, EDB can be established as a safe procedure for the labyrinth and could be considered as the preferred surgical choice for refractory disease. In the challenging treatment of Ménière’s, we have to also focus on the outcome of vestibular preservation, in addition to hearing preservation, since bilateral disease is frequent and can be developed in 30% of cases. The increased administration of ablative procedures puts many patients at a disadvantage if they develop bilateral Ménière’s later in life.

On the other hand, we also evaluated the persistence of aural fullness and tinnitus after 24 months: aural fullness was distributed similarly in our groups (*p* = 0.647) but tinnitus was more prominent in the deteriorated hearing group (*p* = 0.033). This is likely due to the higher damage sustained by hair cells in that group.

### 4.3. Study Limitations and Future Research 

This study has the limitations typically encountered in retrospective reviews as well as has a selection bias imposed by the research question. The impact of selection bias was reduced by including all eligible cases and statistically confirming the homogeneity of the samples. It is important to mention that all surgeries in this study were performed by a single surgeon and that overall good results might not be generalizable to other surgeons and units.

Future research should include long-term follow-up of EDB cohorts to determine the long-term effect of surgery on hearing outcomes, ideally with comparison of matched controls on medical therapy.

## 5. Conclusions

Endolymphatic duct blockage surgery is a safe and effective surgical option for treatment of MD, with excellent vertigo control, hearing preservation or improvement in most cases at 2 years. We could not identify strong predictors of hearing improvement on presentation, but a lower WRS in the interval 40 to 60% may be the best estimator available. Therefore, ablative interventions, such as intratympanic Gentamycin injection, labyrinthectomy or vestibular neurectomy, should be considered very carefully in patients with low WRS, as they may benefit more from EDB; there is a fair chance of a good hearing outcome with EDB. Vertigo control and hearing preservation are independent outcomes of EDB surgery, making it desirable as an early intervention for refractory MD cases.

## Figures and Tables

**Table 1:** Numeric scale value for vertigo based on AAO-HNS [28].

Numeric Value	Control Level	Class
0	Complete control	A
1–40	Substantial control	B
41–80	Limited control	C
81–120	Insignificant control	D
>120	Worse	E
Secondary treatment initiated due to disability from vertigo		F
Formula	$\frac{Average\;spells/month\;post\text{-}treatment\;(24\;months\;recommended)\;\times \;100}{Average\;spells/month\;pre\text{-}treatment\;(6\;months\;recommended)}$	

**Table 2:** Demographics and hearing outcomes at 24 months.

*n* = 66	Deteriorated Hearing 19.7% (*n* = 13)	Stable Hearing 57.6% (*n* = 38)	Improved Hearing 22.7% (*n* = 15)	*p* Value
Age (years)	50.7 [45.9–55.6]	56.7 [51.7–61.8]	53 [47.8–58.2]	0.22
Sex (% Males)	43	29	50	0.343
Preop WRS (%)	71.5 [62.6–80.4]	64.8 [51–78.5]	51.8 [41.1–62.5]	0.032 *
Postop WRS (%)	45.8 [35.2–56.4]	63.4 [50.4–76.5]	80.1 [72.4–87.8]	<0.001 *
Mean WRS gain (%)	−25.7	−1.4	+ 28.3	
Preop PTA (dB)	48.8 [42.8–54.8]	46.3 [37.8–54.9]	54.8 [47.2–62.4]	0.523
Postop PTA (dB)	69.4 [61.9–76.9]	49.1 [39.9–58.4]	46.2 [37.3–55.1]	<0.001 *
Mean PTA gain (dB)	+20.6	+2.8	−8.6	
Preop BCT (dB)	46.4 [40.1–52.8]	43.5 [35.9–51.2]	50.6 [44.7–56.6]	0.301
Postop BCT (dB)	60.7 [55.1–66.3]	42.9 [33.2–52.6]	39.5 [30.4–48.7]	0.001 *
Mean BCT gain (dB)	+14.3	−0.6	−11.1	

[−]: 95% CI (confidence interval); WRS: word recognition score; PTA: pure tone average; BCT: bone conduction thresholds; dB: decibels; preop: preoperative; postop: postoperative. * Statistically significant.

**Table 3:** Preoperative and postoperative factors at 24 months.

*n* = 66	Deteriorated Hearing 19.7% (*n* = 13)	Stable Hearing 57.6% (*n* = 38)	Improved Hearing 22.7% (*n* = 15)	*p* Value
History of ITS injections (%)	28.6	10.5	9.1	0.236
History of Revision Surgery (%)	19	21.1	22.7	1
Intraoperative ELS integrity (%)	39.1	57	68	0.141
Preop. Vertigo spells (6 months)	28.15 [20–36.3]	18 [11.7–26.1]	32.14 [22.7–41.6]	0.17
Postop. Vertigo Class A (%)	87	76	91	0.38
Postop. Vertigo Class B (%)	13	24	9	
Postop. Vertigo Class: C, D, E and F (%)	0	0	0	
Caloric paresis Improvement (affected ear)	11.4 [−1.4–24.2]	7.25 [−7.9–22.4]	9.84 [−1–20.6]	0.22
Persistence of Tinnitus (%)	100	76	82	0.033 *
Persistence of Aural Fullness (%)	70	71.4	60	0.647

[−: 95% CI (confidence interval); ITS: intratympanic steroid; ELS: endolymphatic sac. * statistically significant; preop: preoperative; postop: postoperative.

## Data Availability

Data available on request due to restrictions (ethical).
